# Cocaine and aortic dissection: the need for collaboration to overcome the underreporting bias

**DOI:** 10.1007/s12024-025-00951-7

**Published:** 2025-01-23

**Authors:** Pascale Basilicata, Antonio Lombardi, Mariagrazia Marisei, Emanuele Capasso, Angela Simonelli, Maria Pieri

**Affiliations:** 1 Department of Advanced Biomedical Sciences, School of Medicine, University of Naples Federico II, Via S. Pansini, 5, Naples, 80131 Italy; 2 Legal Medicine Division, Local Health Authority (ASL) Napoli2Nord, Naples, Italy

**Keywords:** Cocaine abuse, Aortic dissection, Underreporting bias, Forensic toxicology

## Abstract

The dissection of the aorta is a serious and potentially fatal consequence of cocaine use. Nonetheless, the underlying mechanisms and characteristics of this phenomenon remain to be deeply studied. The autopsy case of a 46-year-old white male found irresponsive and unconscious in his house and had a history of abusing cocaine is presented. Autopsy findings showed aortic arch and thoracic aorta of regular calibre, with evidence of a mid-adventitial dissecting aneurysm of the ascending intrapericardial portion of the aorta. Forensic toxicological analyses evidenced a positivity to cocaine and its main metabolite, benzoylecgonine, in all fluids (peripheral blood, bile and urine), liver and brain homogenates. Data also evidenced a positivity to alcohol, confirming a past history of intake. The presented case confirms the connection between cocaine addiction and the risk of aortic dissection, emphasizing the need for increased knowledge about the risks connected to this drug. It is emphasized the need of prompt examination and effective treatment of patients exhibiting cardiac symptoms linked to cocaine use, as well as the need of an accurate anamnesis to evidence use/abuse of other cardiotoxic substances.

**Supplementary Information:**

The online version contains supplementary material available at 10.1007/s12024-025-00951-7.

## Introduction

Cocaine is among the most commonly used illicit drugs in Europe, preceded only by Cannabis derivatives, although prevalence levels and patterns of use differ considerably between countries. According to the European Monitoring Centre for Drugs and Drug Addiction (EMCDDA), about 2.3 million young people between 15 and 34 years (2.3% of this age group) have used cocaine in the last year [1]. Cocaine use results in cardiac toxicity [2–6] and chronic use results in an increased risk of aortic dissection [7–9].

The toxic action that cocaine exerts on the cardio-vascular system still presents aspects to be studied and deepened. On the base of results obtained in a case of fatal aortic dissection in a chronic cocaine user, the possible role of underreporting bias in defining risk for aortic dissection due to the drug’s use is discussed.

## Materials and methods

Certified standard solutions of drugs of abuse used for confirmation analysis in gas and liquid chromatography/mass spectrometry (GC/MS and LC/MS, respectively) were from Cerilliant-Merck (Milan, Italy), BSTFA derivatizing agent from Acros (Morris Plains, NJ, USA), and HPLC grade-solvents from Carlo Erba (Milan, Italy). See supplementary material for details on performed analyses.

### Toxicological analysis

Biological fluids (peripheral blood, urine, and bile) and organ homogenates (from brain and liver) were used for toxicological analyses, according to previously published procedures [10, 11]. ELISA screening tests were initially performed on blood, according to manufacturer’s specifications. All “non negative” results were verified with specific confirmation analyses performed on all biological matrices by GC/MS or LC/MS [12, 13]. For GC/MS analyses all samples were acquired in both *full scan* and Selected Ion Monitoring mode (GC/MS-SIM). The eventual presence of ethyl alcohol or any other volatile chemicals was also verified, by HS-GC/MS analysis. The presence of ethyl glucuronide was verified in LC/MS according to Morini et al. [14].

## Case history

A 46-year-old white male was found at his home by his relatives lying on the floor, unconscious and irresponsive. He was then quickly transported to the E.D. where he arrived in cardio-respiratory arrest and with central cyanosis. The sanitary staff started with resuscitation maneuvers according to the Advanced Life Support protocol. The maneuvers were suspended 20 min later due to the persistence of the asystole, with the declaration of patient’s death. From the testimonial findings acquired during the investigation, it emerged that the subject, while alive, was suffering from arterial hypertension, not treated pharmacologically, and, furthermore, used cocaine and alcohol chronically. The Prosecutor Office arranged for judicial autopsy and forensic toxicological analyses, aimed to elucidate the cause of death.

## Results

### Autopsy findings

The autopsy highlighted the presence of morphologically regular heart (weight 380 g) with intact thin and see-through pericardium, containing brownish liquid at the level of the cardiac lodge. The direct inspection of the cardiac lodge showed the presence of blood (approximately 2.5 L) mixed with large blood clots, suggesting that cardiac tamponade occurred. The heart appeared increased in volume and presented fibrinohaematic envelop. On palpation, the normal firm-elastic consistency of the organ appeared diminished (transverse diameter 12 cm; longitudinal diameter 13 cm; diameter antero-posterior cm 5). The atrioventricular cavities were not dilated, exempt from masses and/or thrombi. Cardiac walls were internally lined with opaque endocardium, with an increase in the subendocardial fibro-adipose component (mainly in the left ventricle). The left ventricle was increased in thickness (1.8 cm). The coronary vessels, inspected along the three main branches as far as possible, appeared elastic and non-strident when cut, showing subintimal lipoidotic deposits resulting in a reduction in vessel calibre of up to a maximum of 50%.

**Fig. 1:**
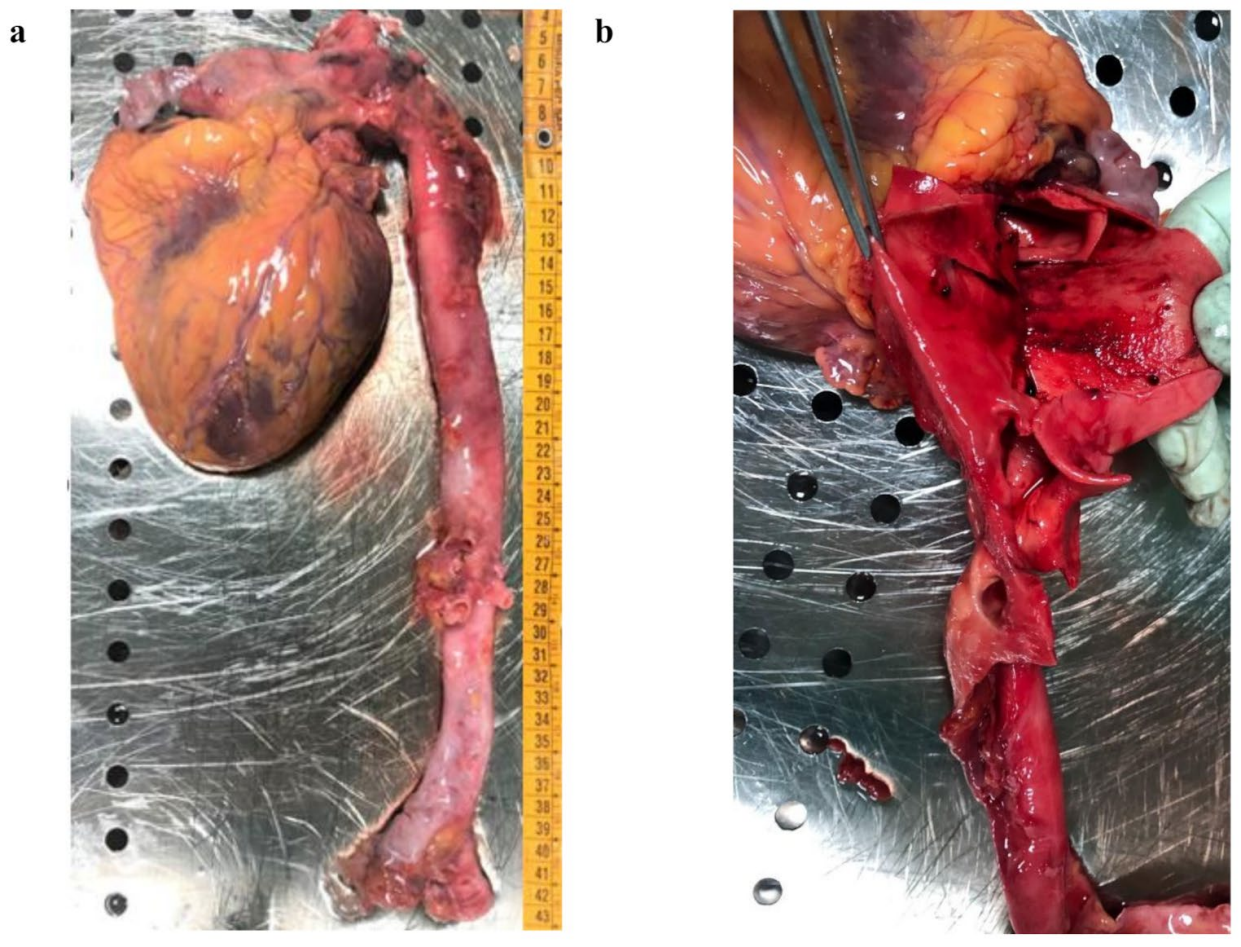
Heart and isolated dissected thoracic aorta (panel **a**). Detail of intimal flap (panel **b**)

Aortic arch and thoracic aorta were of regular calibre (Fig. 1, panel a). Evidence of a mid-adventitial dissecting aneurysm classified as Stanford type A (DeBackey type II) was found at the ascending intrapericardial portion of the aorta (Fig. 1, panel b). Signs of secondary rupture with laceration of the vascular adventitia were identified widely within the described intimal fissure. The dissection chamber, detected approximately 1.5 cm from the emergence of aorta, extended for a length of 14 cm.

All other organs and districts presented normal at external examination.

### Histological analyses

Organ samples were gathered in order to conduct an accurate histopathological investigation. There were several areas of contraction band necrosis, compensatory interstitial hypertrophic cardiomyocytes with enlarged hyperchromatic nuclei, and netlike myocardial fibrosis. Dissecting aorta samples were gathered, and alcian blue, elastic van Gieson, and hematoxylin and eosin (H&E) were used to stain them. Results of histological analyses evidenced the presence of erythrocytes in the adventitia and defect in the muscle layer (*tunica media*).

All other organs and districts were unremarkable.

### Toxicological analysis

Toxicological screening tests performed on an aliquot of the blood sample resulted “non negative” to cocaine and the datum was confirmed by GC/MS. All sampled biological matrices (liquids and organ homogenates) resulted positive to cocaine (COC) and its main metabolite benzoylecgonine (BE). Quantification was performed in GC/MS-SIM, results are presented in Table 1. Blood resulted negative to ethanol; ethyl glucuronide was detected at concentration of 42.0 ng/mL.

**Table 1:** Cocaine (COC) and benzoylecgonine (BE) concentrations detected in analysed biological matrices

Matrix	[COC] (μg/mL)	[BE] (μg/mL)
peripheral blood	132.0	> 1000.0
urine	> 1000.0	> 1000.0
bile	> 1000.0	> 1000.0
	**[COC] (ng/g)**	**[BE] (ng/g)**
brain	782.5	--
liver	897.0	788.7

According to the anatomo-hystological and toxicological evidence, death was attributed to acute aortic dissection, provoked by an acute cocaine intake concomitant to a recent alcohol assumption.

## Discussion

The incidence of acute aortic dissection is not common in clinical practice, and early diagnosis is essential considering that the mortality rate is 25–30% [15–18]. Jannuzzi et al. published a retrospective study using data from the International Registry of Aortic Dissection (IRAD), a retrospective database collecting data of patients diagnosed with aortic dissection worldwide [19].

The abuse of stimulants, especially cocaine, is described in scientific literature as a significant risk factor for the onset of aortic dissection, particularly in young people [20–22]. Cocaine exerts its cardiotoxic effect also at the level of cardiac muscle, leading to various forms of cardiomyopathy [23, 24].

Hsue et al. [21] published a retrospective study based on 20 years’ experience in the field of acute aortic dissection. Results indicate that 37% of patients treated for acute aortic dissection (14 out of a total of 38) reported cocaine use in the minutes or hours immediately preceding the event. Cocaine, especially crack, seems to play a crucial role in aortic dissection in the study group (with a mean age of 41 ± 8.8 years), especially among white individuals (11 out of 14, 79% of total) and those affected by hypertension (11 out of 14, 79% of total). The mortality rate in hospital was 29% (4 patients out of 14).

Dewar and Nolan reported a non-fatal case of aortic dissection in a 34-year-old man who arrived at the emergency department with severe chest pain radiating to the back, accompanied by dyspnea. Cardiac, respiratory, abdominal, and neurological examinations were normal. However, the man admitted cocaine use, with the last assumption three days before the chest pain [25].

From an anamnestic perspective, the here discussed case fits the history of a substance abuse for at least a year when compared to other cases that have been reported in the literature, especially the review by Greve et al. [25]. Authors evidenced as the percentage of tobacco smokers in all reviewed case series ranged from 61 to 100%. Prior research has also revealed a higher incidence of cocaine-related aortic dissection in young male smokers suffering from hypertension [25].

In the presented case the personal history of alcohol consumption is a key element, while no history of smoking is reported, and toxicological analyses resulted negative to nicotine/cotinine. Alcohol is a cardiotoxic substance [24–27] although other research has not reported on such exposure in fatal cases involving aortic dissection in cocaine addicts. The combined use of cocaine and alcohol has been reviewed in relation to violent behavior, but not in fatalities [28], with only one non-fatal reported case of associate heavy consumption of cocaine and alcohol [29]. Apart from the deliberate exposures, it should be mentioned that for the here case discussed, the deceased presented a diagnosis of hypertension and was under pharmacological treatment. However, there was no information available regarding treatment responsiveness, in contrast to another case report in which hypertension was reported under proper pharmacological control [30]. The deceased was diagnosed with a type A aneurysm under the Standford classification, which is the least represented subclassification from the perspectives of cardiac surgery and anatomy-pathology [20–22, 31, 32]. As in the cases already mentioned, the patient here presented was male and fell perfectly within the age ranges of the other case reports in the literature. One of the most interesting aspects to be highlighted in the presented case is the evidence of high urinary cocaine metabolite levels as well as blood concentration, indicating chronic exposure over time to the substance, with a previous residual metabolism of the substance itself. The patient has had a recent alcohol intake, as evidenced by ethyl glucuronate positivity, despite blood alcohol resulted negative. Such evidence, also confirmed by circumstantial datum of a past alcohol abuse reported by his relatives, could have played a key role in determining a vulnerability of the patient to the occurrence of the acute and fatal event.

Regardless of cocaine cardiotoxicity, its possible role in increasing the risk for aortic dissection is still debated, especially due to a lack of epidemiological correlation [25]. Data from case series available in literature [20, 21, 22, 25, 31–32] show features that increase the risk for aortic dissection: young male patients, hypertension, smokers, apart from already mentioned history of cocaine abuse. Difficulties in outlining a correlation between cocaine abuse and aortic dissection on an epidemiological base are mainly related to the low prevalence of AD in cocaine abusers with respect to the general population, accounted for 1.8% (63 cocaine positive out of 3584 patients) according to IRAD data collected from 1996 to 2012 [20]. Cocaine use is associated to the most severe type of aortic dissection according to Stanford classification (B type): data from Dean et al. evidenced a prevalence of 1.4% of cocaine abusers suffering from type A aortic dissection (33/2232), with 2.4% among patients with type B (30/1252) [20]. A reliable explanation of such low prevalence could be a significative underestimation of drug’ use/abuse among patients. Due to the low prevalence of toxicological analyses not often present in diagnostic protocols, data from individual habits are mainly derived from self-reports at clinical history collection. Consequently, the so-called “self-reported bias” could represent a serious issue in limiting the accuracy of the exact prevalence of drugs abuse among patients with AD. A complete toxicological screening should be included in clinical practice, as a unique tool to highlight the real patient status, thus overcoming his/her reticence to self-admit the use. Moreover, the overall differential diagnosis process would benefit, both in terms of prompt and resolutive patients’ management and possible malpractice allegations in case of fatal outcomes. To balance the patient’ right to self-determination and the due to ensure best therapeutic management, an effective information should be offered, with particular emphasis on consequences related to all risk factors, among which cocaine use/abuse could lead to the most fatal outcome, making the patient aware of the real value of a complete and truthful anamnesis. In non-fatal cases the patient awareness about possible future and potentially more severe consequences could positively impact on his/her habits.

The correct estimation of aortic dissection prevalence could benefit from forensic reports about fatal cases not-related to hospitalization. At present such portion of individuals is totally unknown, since such cases are not recorded in IRAD, thus missing critical information in the most severe cases of AD. To cover such a gap, information collected from case reports like the one discussed here could be a starting point for a solid collaboration between clinicians and forensic scientists aimed to comprehensively describe the extent of the phenomenon.

## Conclusion

The case study provides additional support for an anatomopathological association about cocaine toxicity, specifically in relation to the cardiovascular district. This is particularly concerning as it could indicate the highly dangerous possibility of a dissecting aneurysm or aortic dissection.

Data deriving both from clinician settings and forensic activity should be integrated to reach a more accurate and panoptic knowledge of the problem.

## Keypoints

1. The toxic action that cocaine exerts on the cardio-vascular system still presents aspects to be studied and deepened.
2. The autopsy case of a 46-year-old white male with a history cocaine use is presented.
3. The underreporting bias might play a key role in defining risk for aortic dissection in cocaine users.
4. A complete toxicological screening should be included in clinical practice.
5. The correct estimation of aortic dissection prevalence could benefit from forensic reports.

## Electronic supplementary material

Below is the link to the electronic supplementary material.

Supplementary Material 1

## Declarations

### Article classification

10: Forensics

10.010: Pathology

10.040: Toxicology
