# Supplementary material for: Cocaine and aortic dissection: the need for collaboration to overcome the underreporting bias
Source: Forensic Sci Med Pathol. 2025 Jan 23;21(3):1281–6. doi: 10.1007/s12024-025-00951-7 (PMC12491338; doi:10.1007/s12024-025-00951-7)
Supplement: Supplementary file 1 — Supplementary Material 1 [file 12024_2025_951_MOESM1_ESM.docx]

**Supplementary material**

Enzyme Linked ImmunoSorbent Assay (ELISA) screening tests were performed on a Dynex-DSX system from Technogenetics (Chantilly, US), using forensic blood kits from Abbott.

GC/MS analyses were performed using a ISQ single quadrupole mass spectrometer directly linked to a Trace1300 gas chromatograph, all from ThermoFisher (San José, CA, USA). Gas chromatographic separations were performed with a Rxi®-5MS (30m x 0.25mm x 0.25µm) capillary column (Restek, Bellefonte, PA, USA). Data were processed using the Xcalibur software (version 4.0.27.13) (ThermoFisher).

Head-space gas chromatographic/mass spectrometric (HS-GC/MS) analyses were performed on an HP6890 series gas chromatographer provided with a single quadrupole mass spectrometer (Hewlett-Packard); chromatographic separation was accomplished by a CP PorabondQ capillary column (Varian), and data analysed using the MSD Chemstation, software (D.02.0.275 version) from Agilent Technologies.

LC/MS analyses were performed by using an TSQ_TS_ system (ThermoFisher). Chromatographic separation involved the use of a Kinetex® Byphenyl (100 Å, 2.6 µm, 50x2.1 mm) column from Phenomenex (Torrance, CA, US) was used. UHPLC runs involved the following buffers and gradient: Solvent A, 0.1% formic acid in water; Solvent B, 0.1% formic acid in acetonitrile; elution gradient: $5\%B\underset{\to}{0.5min} 5\%B \underset{\to}{1.5 min}30\%B\underset{\to}{2 min} 100\%B\underset{\to}{2.1 min}5\%B\underset{\to}{2 min}5\%B.$ The column was kept at 40 °C, and the flow at 0.3 mL/min. The effluent was directly connected to the electrospray ion source, settled as follows: Positive Ion Voltage 4000 V, Sheath Gas 50 (Arbitrary units), Sweep Gas 1 (Arbitrary units), Ion Transfer Tube Temperature 300 °C, Vaporizing Temperature 350 °C. Data were acquired and processed by using Xcalibur software (2.0.7 version) from ThermoFisher.
